# Carrot Consumption Frequency Associated with Reduced BMI and Obesity through the SNP Intermediary rs4445711

**DOI:** 10.3390/nu13103478

**Published:** 2021-09-30

**Authors:** Kazuya Fujihara, Shun Nogawa, Kenji Saito, Chika Horikawa, Yasunaga Takeda, Kaori Cho, Hajime Ishiguro, Satoru Kodama, Yoshimi Nakagawa, Takashi Matsuzaka, Hitoshi Shimano, Hirohito Sone

**Affiliations:** 1 Department of Internal Medicine, Niigata University Faculty of Medicine, Niigata 951-8510, Japan; kafujihara-dm@umin.ac.jp (K.F.); mr2.yac@gmail.com (Y.T.); yokokawa0323@gmail.com (K.C.); power@med.niigata-u.ac.jp (H.I.); ybbkodama@gmail.com (S.K.); 2 Genequest Inc., Tokyo 108-0014, Japan; nogawa@genequest.jp (S.N.); sk@genequest.jp (K.S.); 3 Department of Health and Nutrition, Faculty of Human Life Studies, University of Niigata Prefecture, Niigata 950-8680, Japan; horikawa@unii.ac.jp; 4 Department of Research and Development, University of Toyama Institute of Natural Medicine, Tomaya 930-8555, Japan; ynaka@inm.u-toyama.ac.jp; 5 Department of Internal Medicine, University of Tsukuba School of Medicine, Tsukuba 305-8577, Japan; t-matsuz@md.tsukuba.ac.jp (T.M.); hshimano@md.tsukuba.ac.jp (H.S.)

**Keywords:** carrot intake, body mass index, single nucleotide polymorphism, rs4445711

## Abstract

It is unclear whether genetic interactions are involved in the association between vegetable intake and reduced body mass index (BMI) or obesity. We conducted a comprehensive search for single nucleotide polymorphisms (SNPs) which are associated with the interaction between vegetable intake frequency and BMI or obesity. We performed a genome-wide association analysis to evaluate the genetic interactions between self-reported intake of vegetables such as carrot, broccoli, spinach, other green vegetables (green pepper and green beans), pumpkin, and cabbage with BMI and obesity, which is defined as a BMI ≥ 25.0 kg/m^2^ in the Japanese population (*n* = 12,225). The mean BMI and prevalence of obesity was 23.9 ± 3.4 kg/m^2^ and 32.3% in men and 22.1 ± 3.8 kg/m^2^ and 17.3% in in women, respectively. A significant interaction was observed between rs4445711 and frequency of carrot intake on BMI (*p* = 4.5 × 10^−8^). This interaction was slightly attenuated after adjustment for age, sex, alcohol intake, smoking, physical activity and the frequency of total vegetable intake (*p* = 2.1 × 10^−7^). A significant interaction was also observed between rs4445711 and frequency of carrot intake on obesity (*p* = 2.5 × 10^−8^). No significant interactions that were the same as the interaction between frequency of carrot intake and rs4445711 were observed between the intake frequency of broccoli, spinach, other green vegetables, pumpkin or cabbage and BMI or obesity. The frequency of carrot consumption is implicated in reducing BMI by the intermediary of rs4445711. This novel genetic association may provide new clues to clarify the association between vegetable intake and BMI or obesity.

## 1. Introduction

Vegetables are an important part of a healthy eating pattern and provide sources of many nutrients. Vegetable intake may be a key modifiable factor in the prevention of chronic diseases such as diabetes, hypertension, and cardiovascular disease [1,2]. Being overweight or obese represents a major public health problem worldwide [3,4,5]. Obesity is an important contributing factor to the global incidence of diabetes, cardiovascular disease, and cancer, leading to a reduced quality of life [6,7]. So far, there has been no consistent finding on the association between vegetable consumption and body mass index (BMI) or obesity [8,9,10,11,12,13,14,15]. While several studies have shown that the intake of some vegetables is negatively associated with BMI [9,10,11,12,13,16], some studies have reported that there was no association between vegetable intake and a subsequent decrease in BMI [12,14,15]. Genetic factors have been reported to be associated with the consumption of vegetables. Matoba et al. showed that TAS1R3 (rs307355; known to be sensitive to the Umami taste) was associated with the consumption of vegetables in the Japanese population [17]. In addition, Calancie and Mikołajczyk-Stecyna et al. reported that TAS2R38 (rs713598, rs1726866, and rs10246939) and gustin (CA6; rs2274333) polymorphisms may affect the frequency of consumption of bitter-tasting foods [18,19].

A recent genome-wide association study (GWAS) revealed many loci associated with adult BMI, and these loci accounted for 2.7% of BMI variation [20]. Pathways involving synaptic plasticity and glutamate receptor activity were pathways that responded to changes in feeding and fasting and are regulated by obesity-related molecules such as BDNF and MC4R [20]. These findings indicate the importance of gene–environment interactions, such as genetic variations and dietary factors; however, it remains unclear whether a genetic interaction role exists between vegetable intake frequency and BMI or obesity. 

Therefore, we conducted a comprehensive search for single nucleotide polymorphisms (SNPs) which interact with the association between various types of vegetables, such as carrot, broccoli, spinach, other green vegetables (green pepper, green beans), pumpkin, and cabbage, and reduced BMI or obesity using a GWAS in Japanese individuals.

## 2. Methods

### 2.1. Study Design

The study participants were customers of the Japanese Direct-to-Consumer genetic testing service, HealthData Lab, provided by Genequest Inc. (Tokyo, Japan) and Yahoo! Japan Corporation (Tokyo, Japan). All participants were over 18 years of age, had answered an online self-reported survey, and had consented to the use of their genotype and questionnaire data for this study. Criteria for inclusion were: (i) aged 18–90 years and (ii) participants of Yahoo HealthData Lab. Criteria for exclusion were those: (i) who had not provided informed consent, (ii) had an incomplete questionnaire or had not provided data, (iii) who were estimated to have non-Japanese ancestry, (iv) who had low call rates per subject, or those with a closely related subject. The final analysis was performed using 12,225 participants. Written informed consent was obtained from all participants. The study purpose was explained to the participants and a further agreement was obtained allowing participants to opt-out. The ethics committee of Niigata University and Genequest Inc. approved the present study (2015-2623 and G2017-0023, 2017-15-1).

### 2.2. Frequency of Vegetable Intake Measurement and Definition of BMI

Frequency of vegetable intake was assessed using an online survey that asked participants “How frequently do you eat carrot, broccoli, spinach, other green vegetables (green pepper and green beans), pumpkin, and cabbage?” The answers included eight categories of intake level: “hardly eat,” “1 to 3 times per month,” “1 to 2 times per week,” “3 to 4 times per week,” “5 to 6 times per a week,” “once per day,” “twice per day,” or “≥ 3 times per day.” Categories were converted into continuous variables (1 to 8), representing frequency, i.e., “hardly eat” was coded as 1, “1 to 3 times per month” as 2, “1 to 2 times per week” as 3, “3 to 4 times per week” as 4, “5 to 6 times per week” as 5, “once per day” as 6, “twice per day” as 7, and “≥ 3 times per day” as 8. In addition, self-reported information on height and weight was collected from all participants. BMI was calculated by dividing weight (kg) by the square of height (m). Obesity was defined as a BMI ≥ 25.0 kg/m^2^, according to the Japan Society for the Study of Obesity [21].

### 2.3. DNA Sampling, Genotyping and Quality Control 

Saliva samples were collected using the Oragene DNA (OG-500) Collection Kit (DNA Genotek Inc., Ottawa, ON, Canada), followed by the extraction of genomic DNA according to the manufacturer’s instructions. Genotyping was performed using either of two Illumina (San Diego, CA, USA) platforms: the HumanCore-12+ Custom BeadChip or the HumanCore-24+ Custom BeadChip. Since these two platforms were designed to measure almost identical marker sets, we used 285,387 markers genotyped by both platforms. We excluded: (i) those estimated to have non-Japanese ancestry [22,23], (ii) those with low call rates per subject (<0.95), (iii) those with a closely related subject (PI_HAT > 0.1875), (iv) those with inconsistent sex data between questionnaire and genotype, (v) SNP markers with low call rates per SNP (<0.95), (vi) values with significant deviation from the Hardy-Weinberg equilibrium (exact test *p* values < 1 × 10^−6^), and/or low minor allele frequencies (<0.01).

### 2.4. Genome-Wide Association Study (GWAS)

We conducted a comprehensive search for SNPs that interact with the intake of various vegetables associated with BMI using PLINK (version 1.90b3.42) [24,25].

First, the covariates included age and sex (regression formula: BMI = α + β_SNP_*SNP + β_age_*age + β_sex_*sex+ β_vegetable intake frequency_*vegetable intake frequency + β_interaction_*(SNP*vegetable intake frequency)). We then added alcohol intake, smoking, physical activity and the frequency of total vegetable intake as covariates. Manhattan and quantile–quantile plots were created using the R software package qqman [26] (version 0.1.4). For SNPs that reached significance, we created regional association plots using LocusZoom [27] (version 1.3). A *p* value < 1 × 10^−5^ was considered as suggestive of significance and a *p* value < 5 × 10^−8^ was regarded as having genome-wide significance. 

## 3. Results

A total of 12,225 participants were included in this study (Table 1). The mean BMI was 23.9 ± 3.4 kg/m^2^ and 22.1 ± 3.8 kg/m^2^ for men and women, respectively. The prevalence of obesity was 32.3% and 17.3% for men and women, respectively. The mean BMI in individuals with obesity was 27.8 kg/m^2^ and 28.5 kg/m^2^ for men and women, respectively. High intake frequency of carrot, broccoli, spinach, other green vegetables (green pepper and green beans), and pumpkin was negatively associated with BMI (*p* = 3.7 × 10^−8^, 1.2 × 10^−3^, 1.2 × 10^−9^, 2.8 × 10^−3^, 2.0 × 10^−11^, respectively); no association was observed for cabbage. 

### 3.1. Interaction between rs4445711 and Frequency of Carrot Intake on BMI

**Figure 1:**
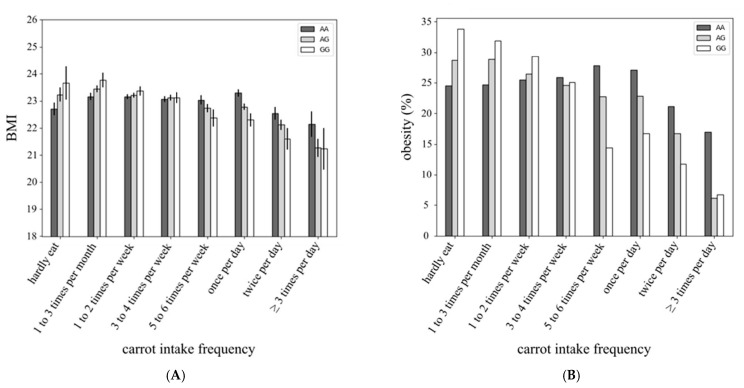
Interaction between rs4445711 and frequency of carrot intake on body mass index (BMI) (**A**) and obesity (**B**) in both men and women.

**Figure 2:**
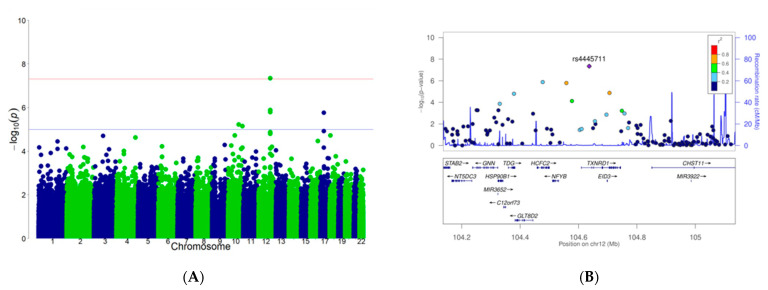
(**A**) Manhattan plot analysis. The *x*-axis represents chromosomal positions and the *y*-axis represents −log_10_ *p* values. The red and blue horizontal lines indicate the genome-wide significance (*p* = 5.0 × 10^−8^) and suggestive significance (*p* = 5.0 × 10^−5^) levels, respectively. (**B**) Area plot of chromosome 12, showing the relative location of rs4445711.

**Figure 3:**
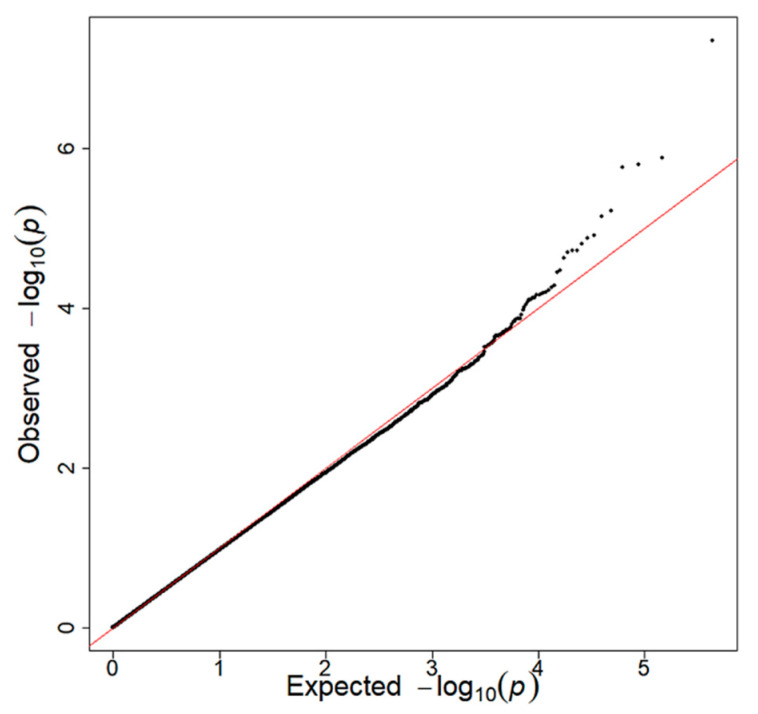
A quantile–quantile plot for the genome-wide meta-analysis showing the *p* value distribution. The *x*-axis represents theoretical −log_10_ *p* values, and the *y*-axis represents observed −log_10_ *p* values. The red line indicates *y* = *x*.

SNPs with suggestive levels of association (*p* < 1 × 10^−5^) with BMI for each vegetable are shown in Table 2. The GWAS revealed a significant interaction between rs4445711 and frequency of carrot intake on BMI and obesity (Figure 1A, *p* = 4.5 × 10^−8^; Figure 1B, *p* = 2.5 × 10^−8^, respectively) adjusted for age and sex. This interaction was slightly attenuated after adjustment for alcohol intake, smoking, physical activity and the frequency of total vegetable intake on BMI (*p* = 2.1 × 10^−7^). Genotype analysis mapped rs4445711 to an intron of the thioredoxin reductase 1 (TXNRD1) gene on human chromosome 12 (Figure 2A), which codes for an antioxidant enzyme regulated by the Nrf2/Keap1 pathway. Figure 2B represents the area plot of chromosome 12 showing the relative location of rs4445711. The overall inflation factor (λ) for the association analysis was 0.9609 (95% confidence interval: 0.9510–0.9705), suggesting a minimal level of confounding with population stratification (Figure 3). There was no association between each rs4445711 allele (AA, AG, GG) and frequency of carrot intake (*p* = 0.80). In addition, no interactions that are the same as the interaction between frequency of carrot intake and rs4445711 were observed between the frequency of broccoli, spinach, other green vegetables (green pepper and green beans), pumpkin, or cabbage intake and BMI or obesity (Supplemental Table S1). The β coefficient for rs4445711 on BMI in the groups which ate carrot 5 or more times per week or less than 5 times per week was −0.438 and 0.128, respectively. No significant association was observed between rs4445711 variants and underweight (*p* = 0.49).

### 3.2. Subgroup Analysis

**Figure 4:**
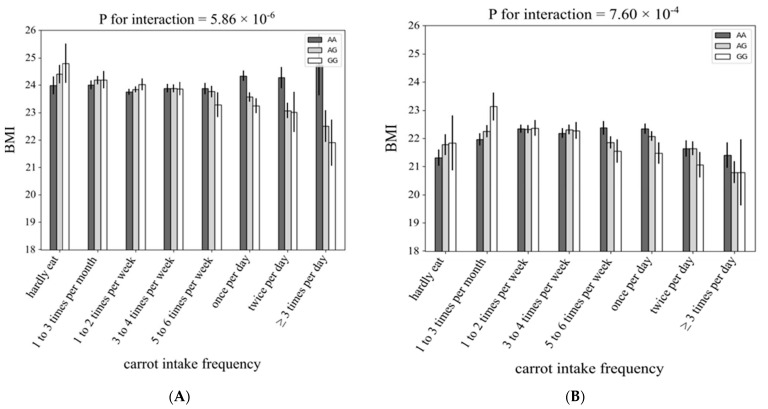
The association between each rs4445711 allele and frequency of carrot intake: subgroup analysis stratified by sex. The *y*-axis represents body mass index according to the genotype of the lead variant at rs4445711—i.e., GG, AG, or AA—and the *x*-axis shows the 8 categories of carrot intake frequency, (**A**) male, and (**B**) female.

**Figure 5:**
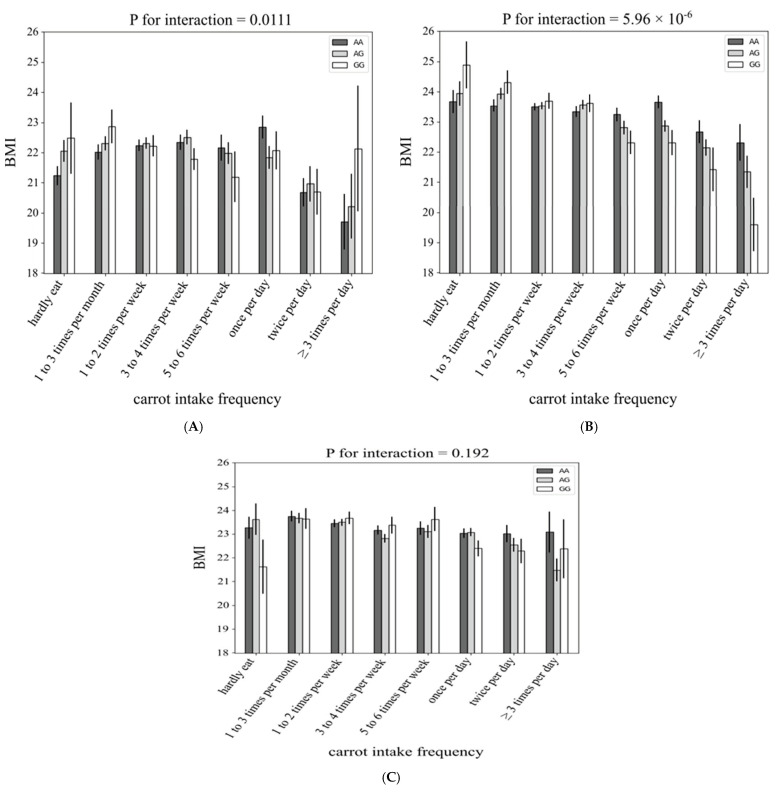
The association between each rs4445711 allele and frequency of carrot intake: subgroup analysis stratified by age. The *y*-axis represents body mass index according to the genotype of the lead variant at rs4445711—i.e., GG, AG, or AA—and the *x*-axis shows the 8 categories of carrot intake frequency, (**A**) younger (≤39 years), (**B**) middle aged (40–59 years), and (**C**) older adult (≥60 years).

Figure 4 and Figure 5 show the association between each rs4445711 allele and frequency of carrot intake in men and women and each age category, respectively. The interaction between rs4445711 and BMI was consistent between men and women (*p* values for interactions in male and female groups were 5.9 × 10^−6^ and 7.6 × 10^−4^, respectively). However, across the age groups, the *p* values for interactions in the younger (≤39 years), middle aged (40–59 years), and older adult (≥60 years) groups were 0.0111, 6.0 × 10^−6^, and 0.192, respectively; no significant interaction was observed in the older adult group.

## 4. Discussion

This is the first report of a comprehensive search for SNPs which associate with the interaction between vegetable intake and BMI or obesity using a GWAS of the Japanese population. 

We showed interactions between frequency of carrot intake and the rs4445711 variant on BMI and obesity. Our data indicated that the G allele of rs4445711 was associated with lower BMI or obesity in individuals with higher frequency of carrot intake.

There have been no consistent reports on an association between vegetable consumption and subsequent decrease in BMI. Wall et al. reported that children who ate vegetables three or more times per week had a lower BMI compared to children who never or occasionally reported eating vegetables [13]. However, no association was observed between vegetable intake in children and the risk of becoming overweight/obese in Western countries [14,15]. In addition, Charlton et al. suggested that improving adherence to dietary targets for fruits and vegetables may be a dietary strategy to overcome overweight among men but not among women [12]. The results indicate the importance of gene–environment interactions, such as genetic variations and frequency of specific vegetable intake. As far as we know, this is the first study to determine the role of interactions between specific genetic factors and the frequency of specific vegetable intake on BMI or obesity. The interaction between frequency of carrot intake and BMI may be dependent on rs4445711; suggesting that carrot intake may contribute to BMI values through rs4445711.

Carrots are rich in carotenoid antioxidants, such as β-carotene, α-carotene and β-cryptoxanthin. Epidemiological studies have shown that a high content of β-carotene in the blood correlates with a low incidence of type 2 diabetes, cancers and mortality [28,29,30,31,32]. Serum β-carotene has also been inversely associated with systemic markers of inflammation and insulin resistance [33,34]. A small-sized cross-sectional study showed that higher intakes of cryptoxanthin was related to low BMI in middle-aged Japanese women [35]. A longitudinal study showed that changes in serum β-cryptoxanthin levels were inversely correlated with changes in BMI in Western countries [36] and Iwata et al. reported that a β-cryptoxanthin-containing beverages produced a greater decrease in BMI and visceral fat area compared with a placebo beverage in pre-obese Japanese men [37]. These results indicate that increased carrot intake may lead to an increase in nutrients which interact with the rs4445711 G-allele. As a result, increased frequency of carrot intake may enhance the reduction in insulin resistance. Future studies are necessary to clarify the effects of serum levels of α-carotene, β-carotene, β-cryptoxanthin and systemic markers of inflammation and insulin resistance on the association between the rs4445711 G-allele and BMI.

The SNP rs4445711 is located in an intron of thioredoxin reductase 1 (TXNRD1) [38], an antioxidant enzyme regulated by the Nrf2/Keap1 pathway. Although rs4445711 has not been reported in the GWAS catalog, other SNPs on TXNRD1 have potential associations with free IGF-1 (rs11112046, *p* = 3.34 × 10^−6^) [39,40,41]. In addition, TXNRD1 levels in adipose tissue correlated with BMI [42]. A previous study showed that rs4445711 associated with the expression of E1A-like inhibitor of differentiation 3 (EID3) and thymine DNA glycosylase (TDG) in subcutaneous fat using eQTL analysis (*p* = 9.4 × 10^−19^, *p* = 2.4 × 10^−6^) [43]. The expression of EID3 was varied in other tissues [44]. Although further research is needed to understand the association between these results and our findings, our results may provide a clue to the mechanism of individual differences in BMI with regard to vegetable intake.

Our study has several limitations. First, we have no data on total carrot intake and carrot cultivars. Second, BMI data were self-reported via the internet, which may have introduced measurement error; however, the accuracy of internet-based BMI is reported to be high [45]. It is important to note that the definition of obesity differs between Japan and Western countries. Third, people who eat more carrots may have other healthy lifestyle behaviors; however, the results did not change even after adjusting for the effects of other vegetable intakes and exercise habits. Forth, although we have made efforts to adjust for factors that might affect BMI, residual confounding factors may exist, such as total calories, macro-nutrients, and dietary fiber intake. Fifth, we did not collect serum carotenoid concentration.

In conclusion, the frequency of carrot consumption has been implicated in reducing the BMI through the intermediary of rs4445711. This novel genetic association may provide new clues to clarify the association between vegetable intake and BMI.

## Figures and Tables

**Table 1:** Characteristics of study participants and frequency of vegetable intake.

Characteristics	Men	Women
Number of participants (*n*)	6495	5730
Age (year)	51 ± 13	50 ± 13
Body mass index (kg/m^2^)	23.9 ± 3.4	21.1 ± 3.8
Obesity (*n*, %)	2097 (33)	994 (17)
Carrot (*n*, %)		
hardly eat	321 (5)	267 (5)
1 to 3 times per month	1204 (19)	790 (14)
1 to 2 times per week	2216 (34)	1569 (27)
3 to 4 times per week	1257 (19)	1172 (20)
5 to 6 times per week	500 (8)	602 (11)
once per day	777 (12)	858 (15)
twice per day	183 (3)	370 (6)
≥3 times per day	37 (1)	102 (2)
Broccoli (*n*, %)		
hardly eat	1140 (18)	761 (13)
1 to 3 times per month	2491 (38)	2295 (40)
1 to 2 times per week	1992 (31)	1777 (31)
3 to 4 times per week	556 (9)	565 (10)
5 to 6 times per week	153 (2)	160 (3)
once per day	147 (2)	147 (3)
twice per day	11 (0)	18 (0)
≥3 times per day	5 (0)	7 (0)
Spinach (*n*, %)		
hardly eat	321 (5)	267 (5)
1 to 3 times per month	1204 (19)	790 (14)
1 to 2 times per week	2216 (34)	1569 (27)
3 to 4 times per week	1257 (19)	1172 (20)
5 to 6 times per week	500 (8)	602 (11)
once per day	777 (12)	858 (35)
twice per day	183 (3)	370 (6)
≥3 times per day	37 (1)	102 (2)
Other green vegetables (green pepper and green bean) (*n*, %)		
hardly eat	629 (10)	368 (6)
1 to 3 times per month	2118 (33)	1542 (27)
1 to 2 times per week	2555 (39)	2273 (40)
3 to 4 times per week	807 (12)	982 (17)
5 to 6 times per week	192 (3)	275 (5)
once per day	164 (3)	246 (4)
twice per day	24 (0)	34 (1)
≥3 times per day	6 (0)	10 (0)
Pumpkin (*n*, %)		
hardly eat	1405 (22)	1020 (18)
1 to 3 times per month	2809 (43)	2587 (45)
1 to 2 times per week	1703 (26)	1454 (25)
3 to 4 times per week	349 (5)	388 (7)
5 to 6 times per week	108 (2)	124 (2)
once per day	100 (2)	129 (2)
twice per day	18 (0)	22 (0)
≥3 times per day	3 (0)	6 (0)
Cabbage (*n*, %)		
hardly eat	148 (2)	118 (2)
1 to 3 times per month	1037 (16)	976 (17)
1 to 2 times per week	2733 (42)	2272 (40)
3 to 4 times per week	1640 (25)	1441 (25)
5 to 6 times per week	446 (7)	483 (8)
once per day	419 (6)	356 (6)
twice per day	57 (1)	68 (1)
≥3 times per day	15 (0)	16 (0)

**Table 2:** SNPs with suggestive levels of association (*p* < 1 × 10^−5^) with body mass index.

CHR	SNP	Position	EA	NEA	BETA	SE	*p*
Carrot							
12	rs4445711	104636601	G	A	−0.1682	0.03073	4.53 × 10^−5^
17	rs223154	29928083	T	G	−0.1445	0.0302	1.73 × 10^−6^
10	rs4919491	95515515	G	A	0.2113	0.0467	6.09 × 10^−6^
10	rs2483855	128933992	A	G	0.1761	0.03924	7.27 × 10^−6^
Broccoli							
4	rs993775	94731641	G	T	−0.1944	0.04095	2.07 × 10^−6^
5	rs13185886	79108476	C	T	0.216	0.04792	6.63 × 10^−6^
Spinach							
None							
Other green vegetables (green pepper and green bean)							
2	rs11692441	156950640	G	T	0.2319	0.05183	7.73 × 10^−6^
2	rs13429725	147292648	G	A	0.186	0.04198	9.44 × 10^−6^
Pumpkin							
3	rs902192	193111865	A	G	−0.3404	0.07406	4.34 × 10^−6^
16	rs9932117	54906895	C	A	0.2347	0.05141	5.01 × 10^−6^
Cabbage							
3	rs12490455	176910577	T	C	−0.2017	0.04464	6.32 × 10^−6^

CHR—chromosome; SNPs—single nucleotide polymorphisms; EA—effect allele; NEA—non-effect allele; BETA—beta-interaction; SE—standard error; *p*—*p*-value.

## Data Availability

All data analyzed during this study are included in this paper and its additional files. Other data are available from the author upon reasonable request.

## Supplementary Materials

The following are available online at https://www.mdpi.com/article/10.3390/nu13103478/s1, Table S1: Interactions between rs4445711 and frequency of various types of vegetable intake on body mass index.

## Abbreviations

BMI	body mass index
EID	E1A-like inhibitor of differentiation
GWAS	genome wide association study
SNPs	single nucleotide polymorphisms
TXNRD	thioredoxin reductase
