# Supplementary material for: Carrot Consumption Frequency Associated with Reduced BMI and Obesity through the SNP Intermediary rs4445711
Source: Nutrients. 2021 Sep 30;13(10):3478. doi: 10.3390/nu13103478 (PMC8538500; doi:10.3390/nu13103478)
Supplement: Supplementary file 1 [file nutrients-13-03478-s001.zip › nutrients-1366881-supplementary.pdf]

Supplemental table S1. Interactions between rs4445711 and the frequency of various types of vegetable intake on body mass index

	CHR	SNP	Position	EA	NEA	BETA	SE	*P*
Carrot	12	rs4445711	104636601	G	A	-0.1682	0.03073	4.53×10^{-8}
Broccoli	12	rs4445711	104636601	G	A	-0.07813	0.04403	0.0760
Spinach	12	rs4445711	104636601	G	A	-0.04899	0.03784	0.195
Other green vegetables	12	rs4445711	104636601	G	A	-0.1326	0.04213	1.66×10^{-3}
Pumpkin	12	rs4445711	104636601	G	A	-0.1271	0.04513	4.86×10^{-3}
Cabbage	12	rs4445711	104636601	G	A	-0.1003	0.04125	0.0151

CHR—chromosome; SNP—single nucleotide polymorphism; EA—effect allele; NEA— non-effect allele; BETA—beta-interaction; SE—standard error; *P*—p-value.
